# Health of Troops at Manila

**Published:** 1899-03

**Authors:** 


### HEALTH OF TROOPS AT MANILA.

On February 2d General Otis reported as follows: Death among troops in Philippines since arrival to February 1st, seven months, 220, of which 41 were due to wounds and accidents. Of the remaining 179, 65 died of typhoid, 43 of smallpox, 22 of dysentery, 8 of malarial fever. The remaining deaths were due to many various diseases. Smallpox causes apprehension. The entire command has been vaccinated several times. Twelve physicians have been engaged several weeks in vaccinating natives. The more sickly season is during the hot months—March, April, May—when fevers, smallpox, and dysentery are more prevalent. Nine per cent. of the command are now reported sick. A great majority of the cases are slight ailments.—*Medical News*.

